# Supplementary material for: SyNDI: synchronous network data integration framework
Source: BMC Bioinformatics. 2018 Nov 6;19:403. doi: 10.1186/s12859-018-2426-5 (PMC6219086; doi:10.1186/s12859-018-2426-5)
Supplement: Supplementary file 5 — Files of the example presented in Additional file 4. This additional file is a zip package that contains all files used in Additional file 4. Detailed descriptions of each of these files are presented in a README file included in this zip package. (GZ 30411 kb) [file 12859_2018_2426_MOESM5_ESM.gz › SyNDITools/Meme2Fimo/meme_4.9.0_4.tar.gz!/meme_4.9.0/doc/scripting.html]

Scripting MEME Web Applications


# Scripting MEME Web Applications

The MEME Suite web applications are exposed as SOAP Web services through the
Opal toolkit.
This means that you can access the MEME suite web applications from
Perl or Python scripts without having to install the MEME Suite locally.

Four sample scripts that demonstrate how to submit jobs to the MEME Web services are provided:

- MemeClient.pl
  demonstrates calling the MEME web application from a Perl script.
- FimoClient.pl
  demonstrates calling the FIMO web application from a Perl script.
- MemeClient.py
  demonstrates calling the MEME web application from a Python script.
- FimoCient.py
  demonstrates calling the FIMO web application from a Python script.

The scripts expect to find two data files in the working directory:

- crp0.fasta
- crp0.meme.xml

The MEME client scripts will run MEME on
crp0.fasta
and store the output in a local directory named `meme_out`.
The FIMO client scripts will run Fimo on
crp0.meme.xml
and
crp0.fasta
and store the output in a local directory named `fimo_out`.

You don't need to have a local copy of MEME Suite installed to
access the web applications from your script, but you do need to have
the appropriate version of the
Opal client libraries
installed.
To install the
Opal client libraries
refer to the
installation Instructions for Perl
or
installation instructions for Python.

The Perl Opal client libraries
require you to have Perl 5.0.8 or higher, and you must have installed
`SOAP::Lite` 0.69 or higher.
You may need to adjust the `PERL5LIB` environment variable to
include the directory containing the Perl Opal client.

The demonstration scripts use the
NBCR MEME Web Server
but you can use a local MEME web server by changing the service URL.
This is specified in the variable `$location` in the Perl script,
and in the variable `serviceURL` in the Python script.

An important advantage of submitting MEME Suite jobs by script is that you
are not limited to the handful of options provided on the HTML forms.
You can specify any of the options available for the command-line version
of the MEME Suite applications.
Note however that the required arguments must come before the optional arguments.
This is the reverse of the standard order when using the command-line directly.
Read the comments in the scripts for more details.

### **Important Note**

Scripting clients can easily submit enough jobs to overwhelm a MEME server,
even one with substantial hardware resources.
If you are sending jobs to a shared server, please submit only one job at a time.
